# Elements of Physiology

**Published:** 1838-08-29

**Authors:** 


### BIBLIOGRAPHICAL NOTICES.

Elements of Physiology. By J. Müller, M. D., Professor of Anatomy and Physiology in the University of Berlin, &c. *Translated from the German, with notes*, by William Baly, Member of the Royal College of Surgeons, and Graduate in Medicine of the University of Berlin. *Illustrated with steel plates and numerous wood engravings. Parts* I., II., *and* III., *containing General Physiology; the Blood and Circulating System; the Lymph and Lymphatic System; Nutrition, Growth, and Re-production; Secretion; Digestion, &c., &c.* London: 1838. 8vo. pp. 848.

This is another and admirable treatise, emanating from, what may be called, the native soil of physiology. Nowhere, from the time of Haller to the present day, has the science of life been cultivated with more untiring zeal, or have more important contributions been made to it, than in Germany. Within a few years, indeed, several systematic works of great merit have appeared from the German press; but these are all still fragmentary, the progress of some having been interrupted by death, or the altered purpose of their author, and the remainder, not yet completed, are now in the course of publication. Although vast genius, labour, and research have been evinced by these investigators of the vital phenomena of the animal economy, their conclusions have too often been warped by prejudice, or disfigured by the ethical subtleties and metaphysical jargon of the schoolman, which has tended very much to impair their actual value, and circumscribe their utility. From these defects the present work is entirely free. It is purely practical. The author, though still in the prime of manhood, has been long known as an indefatigable and zealous cultivator of physiology, and of those branches of science upon which it is based. His numerous invaluable contributions to the periodical literature of his country, besides his larger works on the Physiology of Vision, the Intimate Structure of the Glandular System, and his Anatomy of the Myxinoidæ, have already given him an enviable and merited reputation. Müller is the worthy successor of the celebrated Rudolphi in the physiological chair at Berlin.

The present work, under the unassuming title of Elements of Physiology, exhibits concisely and clearly the actual condition of the science. All the known facts, worthy of record, are given, and the relative value of each, scanned and accorded. Great acuteness and cautiousness, combined with fairness and liberality, are exhibited in the examination of the opinions and theories of the various writers in this department of medicine, and the author's own investigations and their results are mentioned with singular modesty. The importance of an accurate and intimate knowledge of comparative anatomy and physiology, and of organic chemistry to the physiological student, is exhibited in the strongest point of view. They are the beacons by whose light we are to be guided in the discovery of those human phenomena yet unknown. When on the subject of these unsolved processes our author exhibits a commendable abstinence from speculation, candidly pleads ignorance, and clearly and philosophically defines what course will be most likely, by inflexible industry, and suitable inquiry, to lead us to fortunate and correct conclusions. The difficulties of the science are investigated with incredible and untiring perseverance and ability, and such beautiful and simple analyses made of the prominent facts, that were our acquaintance with the principle of life more exact and thorough, the most abstruse and intricate physiological problems would meet with a ready solution. The growth of the science, from the daily accumulation of facts, is so rapid, that with the system here recommended and pursued, we need not despair of beholding, ere long, physiology assuming a definite position among the exact sciences. A considerable portion of the work has been already translated by Mr. Baly, and the three first parts have reached this country. By those who have had an opportunity of comparing it with the original the most unqualified praise has been bestowed. The third part, which has been lately received, contains the completest and clearest history of the present state of the physiology of the nervous system that the general student could desire. The Elements of Physiology should be in the library of every physician in the country, who wishes to possess, in a small compass, the ablest physiological record of the age.

